# *In Vitro* Insulin Resistance Model: A Recent Update

**DOI:** 10.1155/2023/1964732

**Published:** 2023-01-18

**Authors:** Ratih D. Yudhani, Yulia Sari, Dwi A. A. Nugrahaningsih, Eti N. Sholikhah, Maftuchah Rochmanti, Abdul K. R. Purba, Husnul Khotimah, Dian Nugrahenny, Mustofa Mustofa

**Affiliations:** ^1^Department of Pharmacology, Faculty of Medicine, Universitas Sebelas Maret, Jl. Ir. Sutami No. 36A, Surakarta, Central Java 57126, Indonesia; ^2^Department of Parasitology, Faculty of Medicine, Universitas Sebelas Maret, Jl. Ir. Sutami No. 36A, Surakarta, Central Java 57126, Indonesia; ^3^Department of Pharmacology and Therapy, Faculty of Medicine, Public Health and Nursing, Universitas Gadjah Mada, Jl. Farmako, Sekip Utara, Sleman, Daerah Istimewa Yogyakarta 55281, Indonesia; ^4^Department of Anatomy, Histology and Pharmacology, Faculty of Medicine, Universitas Airlangga, Jl Mayjen Prof. Dr. Moestopo 47, Surabaya, East Java 60131, Indonesia; ^5^Department of Pharmacology, Faculty of Medicine, Universitas Brawijaya, Jl. Veteran, Malang, East Java 65145, Indonesia

## Abstract

Insulin resistance, which affects insulin-sensitive tissues, including adipose tissues, skeletal muscle, and the liver, is the central pathophysiological mechanism underlying type 2 diabetes progression. Decreased glucose uptake in insulin-sensitive tissues disrupts insulin signaling pathways, particularly the PI3K/Akt pathway. An *in vitro* model is appropriate for studying the cellular and molecular mechanisms underlying insulin resistance because it is easy to maintain and the results can be easily reproduced. The application of cell-based models for exploring the pathogenesis of diabetes and insulin resistance as well as for developing drugs for these conditions is well known. However, a comprehensive review of *in vitro* insulin resistance models is lacking. Therefore, this review was conducted to provide a comprehensive overview and summary of the latest *in vitro* insulin resistance models, particularly 3T3-L1 (preadipocyte), C2C12 (skeletal muscle), and HepG2 (liver) cell lines induced with palmitic acid, high glucose, or chronic exposure to insulin.

## 1. Introduction

Insulin resistance is the main cause of type 2 diabetes (T2D), which is associated with obesity and metabolic syndrome [1]. In patients with T2D, insulin resistance, which affects insulin-sensitive tissues (e.g., adipose tissues, skeletal muscle, and the liver), is the central pathophysiological mechanism underlying T2D progression [2]. The pathogenesis of insulin resistance is related to free fatty acid (FFA) accumulation, increased liver glucose production, and decreased glucose uptake in insulin-sensitive tissues [3]. Impaired regulation of fatty acid metabolism plays an essential role in the pathogenesis of insulin resistance in the skeletal muscle. Moreover, under insulin resistance conditions, mitochondrial defects in the oxidative phosphorylation process are found in the skeletal muscle [1]. Mitochondria are organelles that produce energy from fat. Hence, a defect in mitochondrial function induces ectopic fat accumulation and leads to the development of insulin resistance. Furthermore, FFA accumulation or hyperglycemic conditions trigger excessive reactive oxygen species (ROS) production in the mitochondria due to decreased mitochondrial function. High levels of ROS further suppress insulin action on the adipose tissues and myotubes as well as inhibit insulin-mediated redistribution of IRS-1 and PI3K, thereby reducing GLUT4 translocation in 3T3L1 cells [4]. Increasing evidence suggests that decreased glucose uptake in insulin-sensitive tissues disrupts insulin signaling pathways [5]. The phosphatidylinositol 3-kinase (PI3K)/Akt pathway has been recognized as an important signaling pathway that mediates the metabolic effects of insulin, with Akt (serine/threonine protein kinase) serving as a key effector molecule [5].

In general, animal models are critical for the development of novel and effective treatments for certain diseases, such as diabetes. Since animals are biologically similar to humans, they are commonly exploited in a variety of diabetes studies [6]. Meanwhile, the use of animal models in preclinical studies is a time-consuming protocol with high costs for maintenance, housing, and breeding. It also raises ethical concerns regarding the unethical protocol of animal handling. Animal rights activists worldwide oppose the use of animals in research, urging researchers to find alternative approaches that can shorten the study while simultaneously reducing the number of animal models used in screening drug candidates. To overcome this limitation, a plethora of new *in vitro* techniques known as “alternative” or “substitute” of animal models have been designed and implemented in drug development studies. The benefits of this replacement include minimizing the use of animal models, obtaining results immediately, lowering research costs, and the possibility of controlling experimental variables [7, 8]. The utilization of *in vitro* cell cultures maintained in a laboratory setting can be a valuable alternative to animal models. The *in vitro* technique is simple and easy to follow. It also takes less time and is less expensive; hence, it could be a potential solution for addressing the 3R principles (reduction, refinement, and replacement) of animal model studies [8]. Although alternative animal models, including the *in vitro* model, have limitations, such as the inability to represent the thorough metabolic response of animals and the limited capacity to determine the pharmacokinetics of drugs or subsequent metabolites, alternative methods, notably *in vitro* models, are required to overcome the limitations of animal models, especially in terms of ethical considerations [7, 8].

An *in vitro* model is suitable for studying the cellular and molecular mechanisms underlying insulin resistance because an *in vitro* model is easy to maintain and the results can be easily reproduced. Cell-based insulin resistance models allow all parameters, including the extracellular environment and culture conditions, to be more precisely controlled and monitored than *in vivo* insulin resistance models. In addition, an *in vitro* model of insulin resistance can avoid tissue crosstalk. Several *in vitro* models of insulin resistance have been applied but with varying inducer exposure. Various insulin resistance inducers can be used *in vitro*, including chronic insulin exposure, dexamethasone, proinflammatory cytokines, adipokines, and hypoxia. Although chronic insulin exposure was most commonly used previously in *in vitro* insulin resistance studies, most *in vitro* insulin resistance models now utilize FFA exposure, especially palmitic acid exposure [9].

A cellular-level study using *in vitro* models is helpful for exploring various aspects of diabetes. Moreover, assessing glucose uptake is still an efficient and reliable approach to measuring the response of biological cells to insulin stimulation [10]. Cell-based models have been widely applied for exploring the pathogenesis of diabetes and insulin resistance as well as for developing drugs for these conditions. However, a comprehensive review of *in vitro* insulin resistance models is still lacking. This review provides a comprehensive overview and summary of the latest *in vitro* insulin resistance models, thereby aiding researchers in determining cell-based models that can be used in the molecular study of insulin resistance. This review is also expected to support the drug discovery of insulin sensitizer candidates.

## 2. Materials and Methods

An extensive literature search was conducted in the PubMed and Scopus databases using keywords such as “*in vitro* model AND insulin resistance,” “palmitate induction AND insulin resistance,” “high glucose induction AND insulin resistance,” “chronic insulin exposure AND insulin resistance,” “C2C12 AND insulin resistance,” “HepG2 AND insulin resistance,” and “3T3-L1 AND insulin resistance.”

From both the abovementioned databases, we identified articles related to *in vitro* models of insulin resistance. Furthermore, the articles were classified based on the *in vitro* insulin resistance model induced by the following: (1) palmitic acid, (2) high concentrations of glucose, and (3) chronic insulin exposure, along with the mechanism of each inducer in triggering insulin resistance. Most articles were published in the last decade and were limited to the English language only.

## 3. Results and Discussion

### 3.1. Cell Lines for Insulin Resistance Model

In scientific research, cell lines have brought a revolution owing to their similarity to primary tissues, low cost, easy application, and simple culture techniques. In addition, cell lines provide an unlimited supply of biomaterials, and their use minimizes the ethical issues associated with the use of animals and humans as study subjects. Cell lines have been widely used in vaccine production, cytotoxicity assays, drug metabolism pathway identification, and therapeutic protein synthesis [11]. Here, we described and summarized several cell lines used as *in vitro* models of insulin resistance.

#### 3.1.1. 3T3-L1 Cell Line

The 3T3-L1 cell line is a well-established preadipose cell line derived from Swiss 3T3 mouse embryos aged 17 to 19 days and displays a fibroblast-like morphology. The 3T3-L1 cell line can exhibit an adipocyte-like phenotype under appropriate conditions and environment [12]. Adipocytes play a central role in the glucose metabolism process [13]. The adipose tissue is also a vital component that responds to insulin stimulation; hence, it can contribute to determining the progression toward insulin resistance [14].

**Figure 1:**
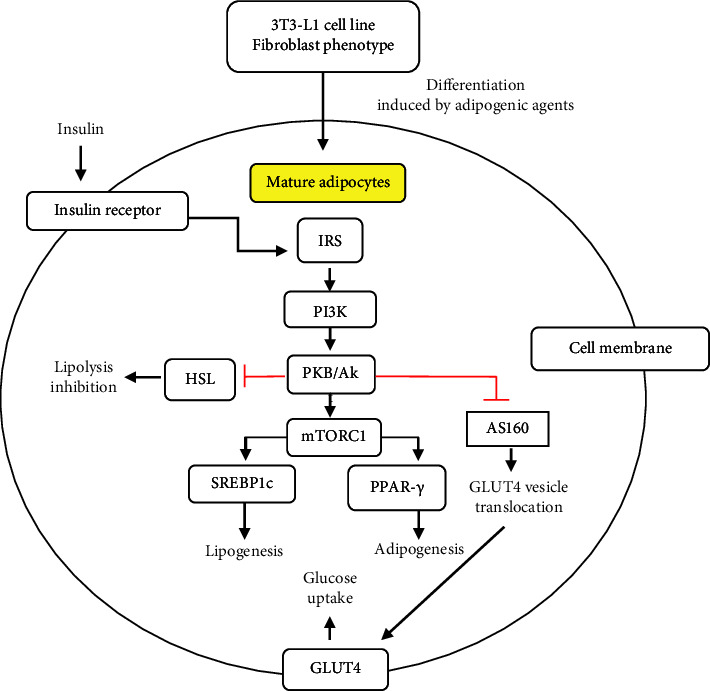
The insulin signaling pathway in adipocytes (mature 3T3-L1 cell lines). Insulin binds to the insulin receptor (IR) and activates the downstream molecules, including IRS1/2, PI3-kinase (PI3K), and Akt. The activated-Akt phosphorylates AS160 and inhibits its activation in stabilizing GLUT4, followed by GLUT4 translocation. It also enhances lipogenesis and adipogenesis via sterol regulatory element binding protein 1c (SREBP-1c) and PPAR*γ* related to mTORC1 activation. Moreover, the activated- Akt inhibits lipolysis by suppressing hormone-specific lipase (HSL).

Insulin plays a pivotal role in various metabolic processes in the adipose tissue, including glucose uptake, *de novo* lipogenesis, adipogenesis, and lipolysis inhibition (Figure 1). Insulin induces glucose uptake in adipocytes through its ability to regulate GLUT4 translocation. Insulin also increases lipogenesis and adipogenesis through SREBP1c and PPAR*γ* stimulation, respectively. In addition, the activity of hormone-sensitive lipase decreases under the influence of insulin, resulting in lipolysis inhibition. In 3T3-L1 cells under basal conditions, 70%–90% of GLUT4 was found in GLUT4 storage vesicles (GSV) [15]. Insulin has been reported to induce 10–20-fold GLUT4 translocation into the membrane cells of 3T3-L1, which are known to fully differentiate into adipocytes [16]. PI3K/Akt pathway-associated AS160 triggers the release of GLUT4 from GSV and its translocation. AS160 is one of the main targets of Akt and plays a critical role in the insulin-stimulated GLUT4 translocation process. AS160 phosphorylation by Akt inhibits its function of stabilizing GSV GLUT4, thereby leading to the release and translocation of GLUT4 [17]. The activation of mTORC1 promotes the induction of SREBP1c and lipogenesis under Akt stimulation [18]. In adipocytes, Akt-associated mTORC1 activation triggers lipogenesis through SREBP1c and promotes adipogenesis through PPAR*γ* [19].

Fully differentiated 3T3-L1 cells (adipocyte cells) serve as a representative *in vitro* model and can be used to comprehend the molecular processes of glucose metabolism [13]. Remarkably, 3T3-L1 cells have been widely implemented as an insulin resistance model, primarily induced by insulin exposure *in vitro* [14]. In general, 3T3-L1 cells with a fibroblast phenotype need adipogenic agents, such as a combination of dexamethasone (DEX), 3-isobutyl-1-methylxanthine (IBMX), and insulin at concentrations of 0.25 *μ*M, 0.5 mM, and 1 *μ*g/mL, respectively, to differentiate into adipocytes. Another study revealed that adding 2 *μ*M of rosiglitazone, besides the combination of the previously mentioned adipogenic agents, successfully induces 3T3-L1 cell differentiation within 10 to 12 days. Moreover, the combination of DEX and troglitazone has been reported to induce differentiation of 3T3-L1 preadipocytes in shorter periods than the combination of IBMX and DEX. Exposure to DEX and troglitazone increases lipid droplet accumulation and glucose uptake mediated by GLUT4 by 112% and 137%, respectively, compared with the differentiation of 3T3-L1 cells induced by traditional methods [12].

Regarding their ease of culture, assay expandability, and reproducibility in differentiation with the ability to reach 100% in optimized conditions, the 3T3-L1 cells have emerged as the most commonly used preadipocyte model for the study of adipocyte development and biology [20]. When compared to freshly isolated mature adipocytes, 3T3-L1 can sustain more passages and have a homogeneous cell population. As a result, these cells respond uniformly to treatments and changes in experimental conditions. Meanwhile, the 3T3-L1 model has certain practical limitations, including the time required for initial subculture as well as the fact that adipogenic differentiation takes at least two weeks. Furthermore, when 3T3-L1 cells reach confluence or have been extensively passaged, they no longer differentiate into adipocytes, are difficult to transfect, and fail to reconstruct the distinctive features of primary cell culture models since this cell line is derived from a single clone [12]. This should be considered when implementing 3T3-L1 as an *in vitro* research model, particularly for metabolic diseases such as diabetes.

Based on the mounting evidence, researchers may decide to use the 3T3-L1 cell line to gain a deeper understanding of the mechanisms underlying adipocytes' insulin resistance, which contributes to the development of type 2 diabetes. In addition, 3T3-L1 cells could be used *in vitro* to develop drugs with specific targets involved in the pathogenesis of adipocyte insulin resistance. To be practical, however, the application of 3T3-L1 should consider the suitable differentiation medium and the time required for the cells' maturation. Additionally, adipogenesis is a temporally dependent process. Therefore, it is essential to comprehend the mechanisms during the various stages of adipocyte differentiation.

#### 3.1.2. C2C12 Myotube Cell Line

In 1977, myoblast C2 cells were first developed to elucidate the molecular mechanism underlying muscular dystrophy *in vitro*. Since then, the C2C12 subclone has been extensively used as a cell culture model for exploring the skeletal muscle [21]. Moreover, C2C12  is an insulin-responsive cell line that serves as a useful model for studying insulin resistance mechanisms. The link between insulin resistance and other metabolic conditions can also be investigated using the C2C12 cell line. It also serves as a model for developing important pharmaceutical compounds and studying drug delivery systems [22].

Among all insulin-sensitive tissues, the skeletal muscle plays a critical role in more than 75% of the glucose utilization process in response to insulin, especially in the postprandial state. Furthermore, insulin resistance in the skeletal muscle is considered an essential condition that influences the development of systemic insulin resistance [23].

**Figure 2:**
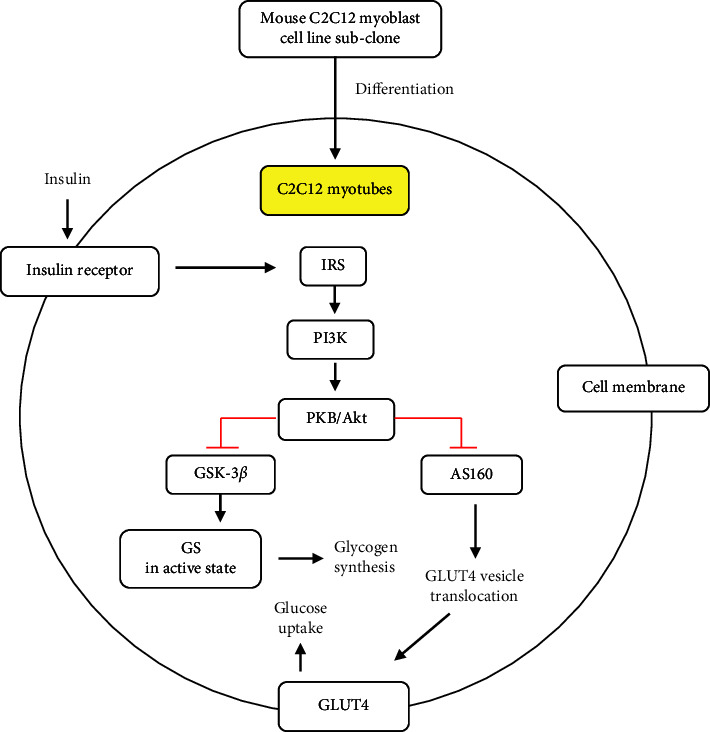
The insulin signaling pathway in skeletal muscles (C2C12 cell lines). The PI3K/Akt activation in skeletal muscle is triggered by insulin binding to its receptor. Activated Akt will promote glucose uptake as well as glycogen synthesis through the inhibition of GSK-3*β* and AS160, respectively.

Insulin mediates skeletal muscle metabolism, especially glucose transport and glycogen synthesis associated with the PI3K/AKT signaling pathway (Figure 2). A series of phosphorylation and activation of PI3K/AKT occurs in the skeletal muscle under insulin-sensitive conditions. Activated Akt can directly phosphorylate and inactivate AS160, thereby triggering the translocation of GLUT4 from GSV to the cell membrane to facilitate glucose uptake. In addition, activated AKT enhances glycogen synthesis in skeletal muscles through GSK-3*β* inhibition. AKT stabilizes glycogen synthase (GS) in an active state to induce glycogenesis. Therefore, the impaired PI3K/AKT signaling pathway will lead to the dysregulation of glucose transport and glycogen synthesis, which are essential for the development of insulin resistance and T2DM. The PI3K/AKT signaling pathway is also considered to occur in the C2C12 myotube, which represents skeletal muscle cells [24].

Along with primary myogenic cells, mouse C2C12 and L6 cells are the most commonly used cellular models for exploring the skeletal muscle *in vitro*. Cell line myoblasts, especially C2C12 cells, are well-established *in vitro* models that have been genetically modified; hence, they can proliferate indefinitely, differentiate into myotubes containing myofibers, and express the contractile protein. The C2C12 and L6 myoblasts can fully differentiate into myotubes expressing insulin-responsive GLUT4, which is critical in facilitating insulin-stimulated glucose uptake in myofibers. Myofibers are the primary site for insulin-induced glucose uptake in the postprandial state. The accumulation of circulating FFA and inflammatory cytokines can directly affect insulin signaling pathways in myofibers [25].

C2C12- and L6-based *in vitro* models exhibited the potential to represent insulin resistance and T2D-related metabolic disorders since these cell lines maintain pivotal insulin signaling pathways, such as IRS-1, PI3K, AMPK, mTOR, and AKT, as well as high levels of GLUT protein expression. *In vitro* exposure of murine myoblasts or myotubes to high glucose or insulin has increased our insight into the mechanisms underlying dysregulation of glucose and insulin in skeletal muscle. This enables us to distinguish between the detrimental effects of abnormally elevated glucose and/or insulin in skeletal muscle from the effects of other T2D pathogenic mechanisms, including fat infiltration and inflammation [26].

C2C12 cells appear as nucleated and spindle-shaped myoblast cells initially. On culturing these cells in suitable media, they undergo cell fusion and form multinucleated structures. C2C12 cells also differentiate rapidly into myotubes, which exhibit elongated fibers after being maintained on differentiation media [22]. Dulbecco's modified Eagle's medium (DMEM) containing glucose, fetal bovine serum (FBS), and antibiotics can be utilized to grow C2C12 myoblasts. After reaching confluence, switching to a low-FBS or horse serum (HS) culture can induce C2C12 differentiation into myotubes [25]. The morphological changes of C2C12 from myoblast into multinucleated myotubes equipped with an actin cytoskeleton have been confirmed via confocal microscopy and immunofluorescence staining [22].

Despite the fact that the widespread use of C2C12 has increased our understanding of T2D pathophysiology, the cell line has limitations in its ability to accurately replicate the complex genetic profile and metabolic dysfunction found in humans [26]. Transcriptomic analysis revealed that C2C12 myotubes and the primary human myotube had both a lower level of genes encoding glucose transporters and a lower oxidative capacity than the L6 myotube. The L6 myotube is probably more suited for exploring glucose metabolism and mitochondrial function, while the C2C12 and primary human myotubes have unique myosin content and glycogen storage. Therefore, they might be better equipped for understanding the exercise/stress response [27].

A critical analysis of the available evidence indicates that primary myotube cultures closely represent the physiology of skeletal muscle. However, its application is limited due to the complexity of the invasive procedures required for its isolation. The L6 or C2C12 myotube cell lines could be used to elucidate the target molecules associated with insulin resistance and the modulation of exercise mechanisms associated with glucose utilization in skeletal muscle in order to understand the molecular pathogenesis and to explore novel drug compounds for T2D. The appropriate selection of L6 and C2C12 models as well as primary myogenic cultures is highly reliant on scientific hypotheses and the biological significance of further study; these facts should be taken into consideration in selecting the proper mature myotubes as an *in vitro* insulin resistance model.

#### 3.1.3. HepG2 Cell Line

Among the various liver cell lines, HepG2 cells first showed the main characteristics of hepatocytes. These cells were isolated in 1975 and defined as hepatoma or hepatocellular carcinoma. In 1980, HepG2 cells were patented as a human hepatoma-derived cell line by researchers at the Wistar Institute. These cells were derived from a well-differentiated hepatocellular carcinoma from the liver of a 15-year-old boy [11].

Being the primary source of endogenous glucose production, the liver is one of the essential insulin target organs. Moreover, it is important for regulating systemic lipid metabolism and mediating the interaction between obesity and T2D. Studying the molecular mechanisms of the relationship between obesity and hepatic insulin resistance pathogenesis and investigating the development of fatty liver disease are significant challenges in advanced biomedical studies [28].

Hepatoma-derived cell lines show specific hepatocyte morphological characteristics and express hepatocyte-specific markers. Therefore, the HepG2 cell line is a more practical alternative than using primary hepatocyte cell culture. The preparation of primary hepatocyte culture needs animal subjects, and the procedure is technically more complex than that of immortalized-cell line culture. In addition, the availability of HepG2 as a human-derived hepatoma cell line is of considerable benefit due to the limited availability of human primary hepatocytes. Thus, the HepG2 cell line is currently being used in several studies involving hepatocyte modeling to study insulin signaling or the molecular mechanisms of metabolic processes [28].

The critical role of hepatocytes in gluconeogenesis has been extensively studied in diabetes because of their dysregulation in insulin resistance conditions. Remarkably, HepG2 cells express glucose-6-phosphatase (G6PC) and phosphoenolpyruvate carboxykinase (PEPCK) as gluconeogenic enzymes [29]. However, the application of HepG2 as an insulin resistance model has several considerations. The inhibition of gluconeogenesis by insulin occurs directly in the liver and indirectly through lipolysis, which decreases the circulation of FFA and glycerol as gluconeogenesis substrates [30]. In addition, an extensive comparative study on insulin signaling that occurs in primary hepatocytes and hepatoma cell lines, especially HepG2, is needed to validate the suitability of using the hepatoma cell line as a model for elucidating insulin signaling and insulin action on glucose metabolism [28].

**Figure 3:**
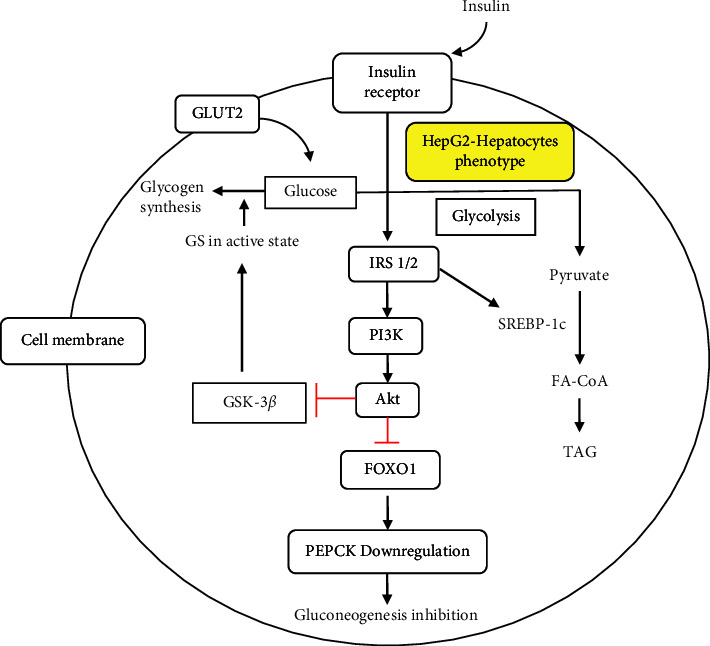
The insulin signaling pathway in hepatocytes (HepG2 cell lines). Insulin binds to the insulin receptor (IR) and activates the downstream molecules, including IRS1/2, PI3-kinase (PI3K), and Akt. The activated Akt will inhibit GSK-3*β* and allow glycogen synthase (GS) to be in an active state to facilitate glycogen synthesis. It also suppresses the transcriptional activity of FOXO1, which will downregulate PEPCK and reduce hepatic glucose production. Insulin enhances *de novo* lipogenesis via sterol regulatory element binding protein 1c (SREBP-1c) to produce triacylglycerol (TAG).

The liver insulin signaling pathway is initiated by the binding of insulin to the insulin receptor (IR), which triggers its activation through the autophosphorylation of tyrosine residues. IR activation triggers the phosphorylation and activation of downstream molecules, such as IRS1/2, PI3K, and Akt/protein kinase B (PKB). The activation of Akt plays a role in inhibiting GSK-3*β* in the inactivation of GS. The active state of GS further triggers glycogen synthesis. Akt activation also inhibits the transcription factor FOXO1 as it induces FOXO1 efflux from the nucleus into the cytosol. This efflux inhibits FOXO1 activity, thereby downregulating PEPCK and inhibiting gluconeogenesis. In addition, some of the glucose from the circulation is taken up by the liver through GLUT2, followed by *de novo* lipogenesis under insulin stimulation [31]. The liver insulin signaling pathway most likely also occurs in the HepG2 cell line that displays the hepatocytic phenotype (Figure 3). Administration of inducers that can disrupt the insulin signaling pathway triggers insulin resistance in HepG2 cells.

Diabetes and its complications may result in pathological alterations in hepatocytes that impair liver function. In contrast, activation of the insulin/PI3K/Akt signaling pathway stimulates glycogen synthesis and inhibits gluconeogenesis in the liver, leading to a reduction in plasma glucose levels [32]. This demonstrates that hepatocytes play a crucial role in the pathogenesis of insulin resistance. Consequently, the HepG2 cell line could provide a valuable tool for identifying drug candidates that target the insulin/PI3K/Akt pathway in the liver.

### 3.2. Inducers of *In Vitro* Insulin Resistance Models and Their Mechanism

#### 3.2.1. Induction of *In Vitro* Insulin Resistance Models Using Palmitic Acid

In recent years, the use of palmitic acid exposure to induce *in vitro* insulin resistance models in several cell lines has become increasingly popular. To investigate the ability of mangiferin to alleviate insulin resistance triggered by the accumulation of free fatty acids, Zhang et al. [33] used a C2C12 and HepG2 insulin resistance model, which was established by exposing C2C12 myotubes or HepG2 to a 0.25 mM palmitic acid solution in a serum-free medium with 1% FFA-free bovine serum albumin (BSA) for 24 hours [33]. Before palmitic acid exposure, the C2C12 myoblast was differentiated first into a C2C12 myotube by switching the medium with 2% horse serum in Dulbecco's modified eagle medium (DMEM) high glucose. The differentiation of C2C12 into a multinuclear myotube takes 7–9 days [33, 34].

Another study used 0.75 mM of palmitic acid to establish an insulin resistance model in the C2C12 myotube. This model was used to investigate Silibinin's activity and molecular mechanism in the inhibition of insulin resistance induced by palmitic acid [35]. Palmitic acid solution was prepared by dissolving palmitic acid with 0.1 M NaOH and heating at 70°C in a shaking water bath. Then, the palmitic acid solution was diluted with 10% FFA-free BSA-DMEM and heated at 55°C to yield a palmitic acid stock solution with a concentration of 5 mM, which was then filtered using a 0.45-mm pore size membrane filter. This solution could be stored at −20°C and used within 2 weeks. The stock palmitic acid solution must be reheated at 55°C for 15 min and cooled to room temperature before use. The exact concentration of NaOH mixed with 10% FFA-free BSA was used as a control [35].

The C2C12 myotube exposed to various saturated and unsaturated fatty acids was used to elucidate the molecular mechanism underlying FFA-related insulin resistance in the skeletal muscle. Moreover, GLUT4 was one of the experimental parameters. A high-fat diet and excessive FFA accumulation in the circulation progressively induce peripheral insulin resistance characterized by the impaired translocation of GLUT4, an insulin-responsive glucose transporter in the skeletal muscle. The negative effect of saturated FFAs, such as palmitate, on the skeletal muscle is related to the abnormal accumulation of several lipid metabolites, such as diacylglycerol and ceramides, resulting in dysregulation of various serine/threonine activations [36].

In a study by Tsuchiya et al. [36], first, after C2C12 myoblasts reached 80%–90% confluency, these cells were differentiated into C2C12 myotubes. The differentiation was conducted by replacing the growth medium with a differentiation medium consisting of DMEM (4.5 g/liter glucose) supplemented with 2% calf serum, 1 nM insulin, 30 *μ*g/ml penicillin, and 100 *μ*g/ml streptomycin. The differentiation medium was changed every 24 hours. Around days 4 and 5, C2C12 cells were entirely differentiated into myotubes and were ready to be used for further experiments. The induction of insulin resistance was initiated by incubating the media containing FFA with DMEM supplemented with 2% FFA-free BSA as described previously [37]. In brief, the FFAs were dissolved in ethanol and diluted in a ratio of 1 : 150 in DMEM containing 2% (w/v) FFA-free BSA and 2% calf serum. The lipid-containing media were incubated for 1 hour at 37°C before being exposed to C2C12 myotubes [36, 37].

Several studies have shown that long-chain saturated fatty acids, including palmitic acid (PA), can disrupt the insulin response, thereby inhibiting glucose uptake in skeletal muscle cells [38]. Various mechanisms underlie the negative effect of high free FFA levels on the skeletal muscle, which causes insulin resistance [23]. PA exposure significantly promotes the phosphorylation of PKC-*θ*, ERK, and proinflammatory cytokines in skeletal myotubes. This process further induces the development of insulin resistance [39].

In addition, incubation of C2C12 myotubes with a high concentration of PA (0.75 mM) for 16 hours has been reported to induce TNF-*α* protein expression, indicating that PA at pathological concentrations may play a role in influencing the overexpression of TNF-*α* as observed in insulin resistance muscle [40]. The insulin resistance induced by TNF-*α* is related to the downregulation of several proteins involved in insulin signaling pathways. The proinflammatory cytokine TNF-*α* has been reported to suppress the IR autophosphorylation response associated with insulin stimulation. Moreover, TNF-*α* induces the Ser/Thr phosphorylation of insulin receptor substrate 1 (IRS-1) while suppressing the insulin-induced Tyr phosphorylation of IRS-1 [41]. In line with this finding, palmitate induction at 0.75 mM has been reported to notably suppress the insulin-stimulated phosphorylation of Tyr^632^ IRS-1 and Ser^473^ Akt. PA also inhibits glucose uptake in insulin-stimulated C2C12 cells by 38% [40]. In addition, PA exposure at a concentration of 0.25 mM was reported to trigger IL-6 expression at both the mRNA and protein levels through a proteasome-dependent mechanism, which further results in the degradation of I*κβ*-*α*, and at the same time, it triggers the activation of NF*κβ* as a critical regulator of proinflammatory signaling [42, 43]. A chronic increase in the activity of proinflammatory signaling pathways in the skeletal muscle and adipose tissues is a critical factor determining the progression of metabolic disorders, including insulin resistance, obesity, and T2D [43].

Another study revealed that the chronic exposure of liver cells to high concentrations of fatty acids, particularly long-chain saturated fatty acids (e.g., PA), disrupts insulin signaling pathways. Eighteen hours of incubation of HepG2 cells and mouse primary hepatocytes with high concentrations of PA have been reported to impair insulin signaling pathways. In HepG2 control cells, acute insulin stimulation triggers tyrosine phosphorylation of insulin receptors (Tyr 1162/1163) and IRS1 (Tyr 612) as well as serine phosphorylation of Akt (Ser 473) in a dose-dependent manner. In contrast, exposure of HepG2 cells to 750 *μ*M palmitate suppresses insulin-stimulated serine phosphorylation of Akt by 65%–70%, which is related to endoplasmic reticulum (ER) stress induction [44]. Mounting evidence has indicated the link between ER stress and insulin resistance through the direct mechanism of insulin signaling pathway disruption [45]. Moreover, an increase in unfolded protein or a conformational change in the ER can trigger the unfolded protein response (UPR), which is a mechanism for coping with ER stress. If the UPR mechanism or increased chaperone protein is not sufficient to cope with ER stress, it will trigger the expression of proapoptotic transcription factors, such as the homologous transcription factor C/EBP (CHOP). Palmitate has been reported to induce the expression of CHOP in liver cells [46].

#### 3.2.2. Induction of *In Vitro* Insulin Resistance Models Using High Glucose Exposure

High glucose concentrations are known to induce cellular damage; this condition is known as glucose toxicity. High glucose concentrations may trigger insulin resistance, especially in the skeletal muscle [47], justifying its use as an inducer in insulin resistance models. A study investigating the molecular mechanism of oxymatrine in the modulation of hepatic gluconeogenesis, including the regulation of PEPCK and G6Pase expression and Akt phosphorylation, used the HepG2 insulin resistance model, which was induced by a high concentration of glucose (55 mM) for 24 hours. The exposure to 55 mM glucose triggers glucose production while suppressing glucose uptake in HepG2 with mechanisms related to Akt inhibition and the upregulation of PEPCK and G6Pase expression. Before treatment, HepG2 cells were cultured in DMEM media supplemented with 10% FBS, 100 U/ml penicillin, and 100 *μ*g/ml streptomycin in a humidified atmosphere of 5% CO_2_ at 37°C; the cells were cultured until they reached 70% confluency [48].

In another study that evaluated the scopoletin effect in ameliorating insulin resistance, Zhang et al. [49] used the HepG2 insulin resistance model, which was induced by high glucose, with Akt phosphorylation as one of the measured parameters. After HepG2 cells reached maximum confluence, they were reincubated for 24 hours in serum starvation media. The cells were further incubated for 24 hours with serum-free DMEM containing either normal (5.5 mM) or high concentrations of glucose (30 mM) with or without 10 *μ*M of scopoletin and/or 5 *μ*M of rosiglitazone as a positive control. In the high-glucose concentration (30 mM) group without insulin or scopoletin addition, there was a significant inhibition of Akt phosphorylation compared to the control group without treatment [49]. Gao et al. [50] treated fully differentiated 3T3-L1 cells with high glucose at a concentration of 25 mM and 0.6 nM insulin for 18 hours to investigate the effect of 4-hydroxyisoleucine on alleviating an insulin-resistant-like state. Those treatments successfully established an insulin resistance model in 3T3-L1, as evidenced by the inhibition of 2-deoxy-[^3^H]-d-glucose uptake and an increase in TNF-*α* [50].

Persistent hyperglycemia induces glucose toxicity and has deleterious effects on various cell types. Irreversible pancreatic *β*-cell damage due to glucose toxicity induced by chronic exposure to high glucose concentrations contributes to the development of insulin resistance and secretory dysfunction. Pancreatic *β*-cell glucotoxicity is mainly related to oxidative stress. Elevated glucose levels trigger ROS production, and the lower capacity of antioxidant enzymes in pancreatic *β*-cells may also render *β*-cells more susceptible to oxidative damage than other cells. High glucose levels interact with proteins and trigger the formation of excessive advanced glycation end products, which enhance oxidative stress. Some of these mechanisms underlie the link among glucose toxicity, ROS accumulation, oxidative stress, and cell dysfunction, which trigger the progression of insulin resistance [51].

#### 3.2.3. Induction of *In Vitro* Insulin Resistance Models Using Chronic Insulin Exposure

Chronic insulin exposure is associated with insulin resistance and has been reported to induce insulin resistance models *in vitro* in several studies. Rossi et al. [14] used 3T3-L1 cells as one of the models to characterize the effects of chronic hyperinsulinemia exposure. Before being used as a model, 3T3-L1 cells need to be differentiated from the fibroblastic phenotype to mature adipocytes by incubating 3T3-L1 cells with a differentiation medium containing 1 *μ*g/mL of insulin, 0.25 *μ*M of dexamethasone, 0.5 mM of IBMX, and 2 *μ*M of rosiglitazone [14, 52]. The differentiation process was observed using Oil-Red-O staining. On the seventh day, intracellular lipid droplets began to appear and increased in size and number with an increase in the incubation time. The whole differentiation process took approximately 14 days [52]. The chronic stimulation of 3T3-L1 used human recombinant insulin at a concentration of 150 nM, which was added from day 6 postdifferentiation induction until the day before the experiments. Insulin exposure and serum starvation should be administered for at least 18 hours before the study. The experiments were parallel for untreated and insulin-treated cells [14].

Another study used C2C12 myotube cells exposed to chronic insulin concentrations of 60 nM for 24 hours to induce an insulin resistance model. Furthermore, in assessing cellular response to insulin stimulation, the C2C12 cells were exposed to 120 nM of insulin for 15 min before the study to activate the signaling pathway [5]. A study to evaluate the effects of ginsenoside Rg1 in triggering glucose uptake in the insulin resistance model was also conducted in the C2C12 cell line induced by chronic insulin exposure. The C2C12 cells were incubated with a growth medium consisting of DMEM, 10% FBS, and 1% antibiotics. After reaching confluence, the C2C12 cells were differentiated and exposed to 100 nM of chronic insulin for 3 days. On day 4, the differentiation of C2C12 cells was continued in DMEM supplemented with 2% HS, while ginsenoside Rg1 was given for the last 12 hours prior to the measurement of the parameters [53].

Feng et al. [54] explored the effect of chromium malate in ameliorating insulin resistance in the 3T3-L1 adipocyte model. Fully adipocyte-mature 3T3-L1 cells were obtained after 2 days of treatment with insulin (10 mg/mL) and IBMX (0.5 mM), followed by 5 days of treatment with 10% FBS-DMEM. For establishing an insulin resistance model, differentiated 3T3-L1 cells (2 × 10^5^ mL) were seeded in 96-well plates with 10% FBS-DMEM and incubated at 37°C for 24 hours, followed by 24 hours of insulin incubation at a concentration of 10^−6^ mol/L. As shown by an increase in glucose levels, chronic insulin exposure triggers insulin resistance in 3T3-L1. This effect is related to the downregulation of the glucose uptake-related pathways (GLUT4, Akt, and p-Akt). In addition, it inhibits insulin-sensitive signaling pathways, including IRS1, PPAR*γ*, PI3K, and p38-MAPK, at both mRNA and protein levels [54].

Insulin resistance in an individual with an adequate *β*-cell function can be compensated via increased insulin secretion by *β*-cells. However, increased insulin secretion can aggravate insulin resistance since an increase in circulating insulin concentration tends to suppress the insulin response. Primary insulinoma patients without a history of metabolic syndrome also develop insulin resistance, which may result from tumor-induced insulinemia. In addition, patients with diabetes receiving pulsatile insulin therapy showed better glucose control than those receiving continuous insulin infusion [55].

Hyperinsulinemia decreases the activity of insulin receptors; hence, it contributes to insulin resistance. After chronic insulin incubation, the autophosphorylation ability of insulin receptors in response to insulin stimulation is restricted [52]. Moreover, hyperinsulinemia can trigger insulin resistance, especially that related to fatty acid accumulation, since inhibition of the IRS1/2 function after the activation of negative feedback in insulin signaling pathways is observed in hyperinsulinemia [56].

This review shows that *in vitro* models focusing on several cell lines (3T3-L1, C2C12 myotube, and HepG2) from adipose tissue, skeletal muscle, and the liver to represent the main loci of impaired insulin action serve a great benefit and simplify the procedure of studying insulin resistance mechanisms (summarized in Table 1).

Despite their numerous benefits, *in vitro* studies have a number of drawbacks that should be considered. Friesen et al. [57] reported that the most critical limitation of *in vitro* cell culture systems is that the nutritional ingredients contained in the culture media do not fully represent the material contained in human tissue plasma *in vivo*. Several attempts have been made to overcome the limitation, including the enrichment of the cell culture media contents to mimic human plasma. However, *in vitro* culture models are still lacking due to specific nutrition at the supraphysiological level or incomplete validation of the insulin signaling response [57].

Another limitation of the *in vitro* model is the human body's complex system; hence, *in vitro* tests alone are not sufficient for studying the whole-body system. Therefore, the effectiveness of drug candidates should also be assessed using an *in vivo* system to gain a comprehensive understanding. *In vitro* models are helpful and recommended for determining a suitable target molecule or receptor at an early stage. However, an *in vivo* model is required and recommended for further research to develop drug candidates and assess their safety profile through toxicological tests [58].

### 3.3. Future Remarks

Over the recent decades, 2D cell culture has been utilized in biological studies, including high-throughput screening (HTS) for small drug libraries. The majority of cell-based HTS is conducted on two-dimensional (2D) cell cultures grown on plastic surfaces customized for tissue culture. Meanwhile, 2D culture is incapable of expressing the microenvironment of *in vivo* biology, which influences the physiology of cells. Previous research has demonstrated the necessity of the extracellular matrix (ECM) on cell behaviour and that 3D cell culture can more accurately represent the *in vivo* environment than 2D cell culture systems. Moreover, conventional 2D culture systems were unable to mimic the inflammatory conditions present during adipocyte differentiation. Integration of inflammatory microenvironments with mature adipocytes is required for the development of drugs targeting metabolic disorders such as obesity and insulin resistance [59]. Interactions occur between cells and cell-matrix shape molecular phenotypes and controlled cell morphology, proliferation, and metabolism. This interaction is disturbed by conventional 2D monolayer culture. To closely represent the cellular microenvironment, 3D cell culture was developed [20].

Multiple factors involving crosstalk between organs and tissues, or even between cellular compartments within the same tissue, are frequently responsible for metabolic system disorders. Numerous physiological stressors might disrupt the tightly regulated mechanism of *β*-cell function in the context of metabolic overload and insulin resistance, both of which are frequently observed in obesity-related T2D in humans. T2D is characterized by a disruption in the crosstalk between two or more organs' homeostatic mechanisms. Nonetheless, it is necessary to investigate the potential platforms for the study of diseases that progress through the perturbation of this crosstalk. Traditional *in vitro* models for the study of organ interactions were limited to coculturing different cell types and transferring media from one cell to another, and they failed to capture the time-dependent dynamics of multiple organ direct interactions. Consequently, models with sufficient complexity are required to recreate their relevant characteristics *in vitro*. Organ-on-a-chip platforms are an attractive candidate tool for the characterization of multifactorial pathologies, including diabetes, allowing the interaction of different organs and tissues. It also simulates systemic interrelationships by coculturing different organs on a chip in separate compartments connected by microfluidic channels. Initially, these systems were used to demonstrate the *in vivo* pharmacokinetic and pharmacodynamic responses of a particular drug. With the establishment of 3D environments and long-term cultures, the concept expanded further into drug metabolism, and successive advancements were made to investigate cell-cell interactions [60].

The further development of *in vitro* T2D models will allow researchers to reverse-engineer the complexities of *in vivo* T2D signaling in an *in vitro* setting. The 3D tissue frameworks will offer novel therapeutic strategies and contribute towards the development of novel T2D therapies with precise mechanisms [26].

## 4. Conclusions

Cell lines have brought a revolution and a shift in scientific study. Cell lines have been used for studying the pathogenesis of diabetes and insulin resistance as well as discovering drugs for these conditions. The utilization of the 3T3-L1 (preadipocyte), C2C12 (skeletal muscle), and HepG2 (liver) cell lines induced by palmitic acid, high glucose, or chronic insulin exposure has been well established in the exploration of diabetes and insulin resistance, considering that these cell lines represent organs that are crucial in the pathogenesis of insulin resistance and T2D progression. Furthermore, *in vitro* models could be a reasonable option for bringing up the 3R principles (reduction, refinement, and replacement) and useful to overcome the limitations of animal models, particularly in terms of ethical considerations.

## Figures and Tables

**Table 1:** A summary of the *in vitro* insulin resistance model.

*In vitro* insulin resistance model	Inducing methods	Important findings	References
C2C12 myotube and HepG2	0.25 mM palmitic acid solution in a serum-free medium with 1% FFA-free bovine serum albumin (BSA) for 24 hours of incubation	(i) The glucose uptake and p-Akt declined significantly on both cell lines (ii) The decrease in expression of GLUT2 and GLUT4 on HepG2 and C2C12 myotubes, respectively (iii) The increasing glucose content in both cell lines	[33]

C2C12 myotube	0.75 mM palmitic acid in 10% FFA-free BSA-DMEM for 16 hours of incubation	(i) The significantly reduced 2-NBDG uptake in insulin-stimulated C2C12 myotubes (ii) The inhibition of GLUT4 translocation (iii) Downregulation of Tyr^632^ phosphorylation of insulin receptor substrate 1 (IRS-1) while upregulation IRS-1 Ser^307^ phosphorylation	[35]

C2C12 myotube	0.75–1 mM palmitic acid in DMEM containing 2% (w/v) FFA-free BSA and 2% calf serum for 16 hours of incubation	(i) Palmitate (C16: 0) inhibits GLUT4 trafficking by inducing sortilin downregulation via a PKC-*θ*-mediated mechanism (ii) It also significantly promotes the TNF-*α* secretion	[36]

C2C12 myotube	750 *μ*M palmitate in DMEM containing 2% FFA-free bovine serum albumin	(i) The increase of PKC-*θ* and ERK phosphorylation (ii) The reduction of 2-deoxy glucose uptake, p-Akt, and GLUT4 translocation	[39]

C2C12 myotube	0.75 mM palmitic acid in DMEM containing 1% FFA-free BSA for 16 hours of incubation	(i) The upregulation of TNF-*α* protein expression (ii) The suppression of insulin-stimulated phosphorylation of IRS-1 and Akt at Tyr^632^ and Ser^473^, respectively (iii) The inhibition of insulin-dependent glucose uptake	[40]

C2C12 myotube	0.25 mM palmitate solution in FFA-free BSA for 20 hours of incubation	(i) Stimulation of both IL-6 mRNA expression and protein secretion by a proteasome-dependent pathway which results in rapid and chronic activation of nuclear factor-*κ*B	[42]

HepG2	750 *μ*M palmitate coupled to BSA for 18 hours of incubation	(i) Lowered insulin-stimulated Akt phosphorylation and glycogen synthesis, along with increased glucose-6-phosphatase expression (ii) This process is related to endoplasmic reticulum (ER) stress induction	[44]

HepG2	A high concentration of glucose (55 mM) for 24 hours	(i) Triggers glucose production while suppressing glucose uptake with mechanisms related to AKT inhibition and the upregulation of PEPCK and G6Pase expression	[48]

HepG2	High concentration of glucose (30 mM) in serum-free DMEM for 24 hours	(i) A significant inhibition of Akt phosphorylation	[49]

Fully differentiated 3T3-L1	High glucose at a concentration 25 mM and 0.6 nM insulin for 18 hours of incubation	(i) The inhibition of 2-deoxy-[^3^H]-d-glucose uptake and an increase in TNF-*α*	[50]

Fully differentiated 3T3-L1	Human recombinant insulin (150 nM), which was added from day 6 of postdifferentiation induction until the day before the experiments	(i) Impairment of insulin signaling (lower p-Akt, p-IRS1, and p-IR)	[14]

C2C12 myotube	Chronic insulin concentrations of 60 nM for 24 hours	(i) The inhibition of insulin signaling, especially p-Akt	[5]

C2C12 myotube	Chronic insulin exposure (100 nM) for 3 days	(i) Lowered 2-deoxy glucose uptake (ii) The inhibition of AMPK pathway	[52]

Fully differentiated 3T3-L1	24 hours of insulin incubation at a concentration of 10^−6^ mol/L	(i) The downregulation of the glucose uptake-related pathways (GLUT4, Akt, and p-Akt) (ii) Inhibition of insulin-sensitive signaling pathways, including IRS1, PPAR*γ*, PI3K, and p38-MAPK, at both mRNA and protein levels	[53]

## Data Availability

The data used to support the results and discussion of this study are included within the article and cited in the references.
